# Catheter-based ablation of supraventricular tachycardias in a paediatric population – a decade of experience at a single centre

**DOI:** 10.1186/2194-7791-1-S1-A29

**Published:** 2014-09-11

**Authors:** Backhoff David, Bahrke Sophia, Kriebel Thomas, Müller Matthias, Schneider Heike, Paul Thomas, Krause Ulrich

**Affiliations:** Department of Paediatric Cardiology and Intensive Care Medicine, University Medical Center Göttingen, Göttingen, Germany

Accessory atrioventricular pathways (AP) and dual AV nodal physiology are common substrates for paroxysmal supraventricular tachycardias (SVT) in children and adolescents. Definitive treatment can be achieved by catheter ablation with either radiofrequency (RF) or cryoenergy. Aim of this study was to assess effectiveness and safety of RF- and cryoenergy ablation in a paediatric population.

During the last 10 years 562 EP studies have been performed in 479 children and adolescents at our institution. Median age was 12.1 yrs (range 0.4 – 19.8). Indications included patient’s preference (67.3%), drug refractory SVT (31.5%) and malignant arrhythmias (1.2%). RF was used in 75%, cryoenergy in 16 %, both energy sources in 8%. No ablation was performed in 1%. Congenital heart disease was present in 39 children (6.9%).

Substrates documented were APs in 54.6% and AVNRT in 42.6%. APs and AVNRT combined were present in 2.8%. APs were interrupted in 90.5% (RF) / 90% (cryoenergy). Dual AV nodal physiology was successfully treated in 98.5% (RF) / 100% (cryoenergy). In 70 patients, repeated EPS due to SVT relapse or recurrent preexitation pattern was necessary. In 3 patients (0.5%) pacemaker implantation due to AV-block after RF ablation was necessary (AVNRT n=2; AP n=1). Routinely performed coronary angiography showed insignificant coronary artery narrowing in 2 patients (0.3%) after RF ablation of right posteroseptal AP. In 1 case (0.2%), thrombosis of the right coronary artery occurred after intercoronary mapping with a 2F catheter. In 2 cases (0.3%), pericardial effusion was evident and required puncture after cryo- and RF- ablation, respectively. Vessel injuries at the puncture site with need for surgical intervention occurred in 6 cases (1%).

Catheter ablation of SVT in children and adolescents was safe and effective. Major complications were noted after RF-application only. Therefore, cryoenergy may be considered as energy source of choice for ablation in the rightseptal area.

